# The effect of insurance status on treatment modality in advanced oral cavity cancer

**DOI:** 10.1186/s40463-022-00608-3

**Published:** 2023-04-18

**Authors:** Shanmugappiriya Sivarajah, Daniel Ghods-Esfahani, Alexandra Quimby, Fawaz Makki, Giacomo Montagna, Hadi Seikaly

**Affiliations:** 1 Department of Otolaryngology-Head and Neck Surgery, Division of Otolaryngology-Head and Neck Surgery, University of Alberta Hospital, 8440 112 St NW, Edmonton, AB T6G 2B7 Canada; 2 Department of Medicine, University of Alberta, Edmonton, Canada; 3 Department of Otolaryngology-Head and Neck Surgery, University of Ottawa, Ottawa, Canada; 4 Department of Surgery, Memorial Sloan Kettering Cancer Center, Breast Service, New York, USA

**Keywords:** Insurance, Treatment, Head and neck cancer, Surgery

## Abstract

**Background:**

Insurance status has been shown to impact survival outcomes. We sought to determine whether insurance affects the choice of treatment modality among patients with advanced (T4) oral cavity squamous cell carcinoma.

**Methods:**

This is a retrospective, population-based cohort study using the Survival, Epidemiology, and End Results Program database. The population included all adult (age ≥ 18) patients with advanced (T4a or T4b) oral cavity squamous cell carcinoma diagnosed from 2007 to 2016. The main outcome was the odds of receiving definitive treatment, defined as primary surgical resection. Insurance status was categorized into uninsured, any Medicaid, and insured groups. Univariable, multivariable, and subgroup analyses were performed.

**Results:**

The study population consisted of 2628 patients, of whom 1915 (72.9%) were insured, 561 (21.3%) had Medicaid, and 152 (5.8%) were uninsured. The multivariable model showed that patients who were 80 years or older, unmarried, received treatment in the pre-Affordable Care Act (ACA) period, and who were on Medicaid or uninsured were significantly less likely to receive definitive treatment. Insured patients were significantly more likely to receive definitive treatment compared to those on Medicaid or uninsured (OR = 0.59, 95% CI 0.46–0.77, *p* < 0.0001 [Medicaid vs. Insured]; and OR = 0.48, 95% CI 0.31–0.73 *p* = 0.001 [Uninsured vs. Insured]), however these differences did not persist when considering only those patients treated following the 2014 expansion of the ACA.

**Conclusions:**

Insurance status is significantly associated with treatment modality among adults with advanced stage (T4a) oral cavity squamous cell carcinoma. These findings support the premise of expanding insurance coverage in the US.

**Graphical Abstract:**

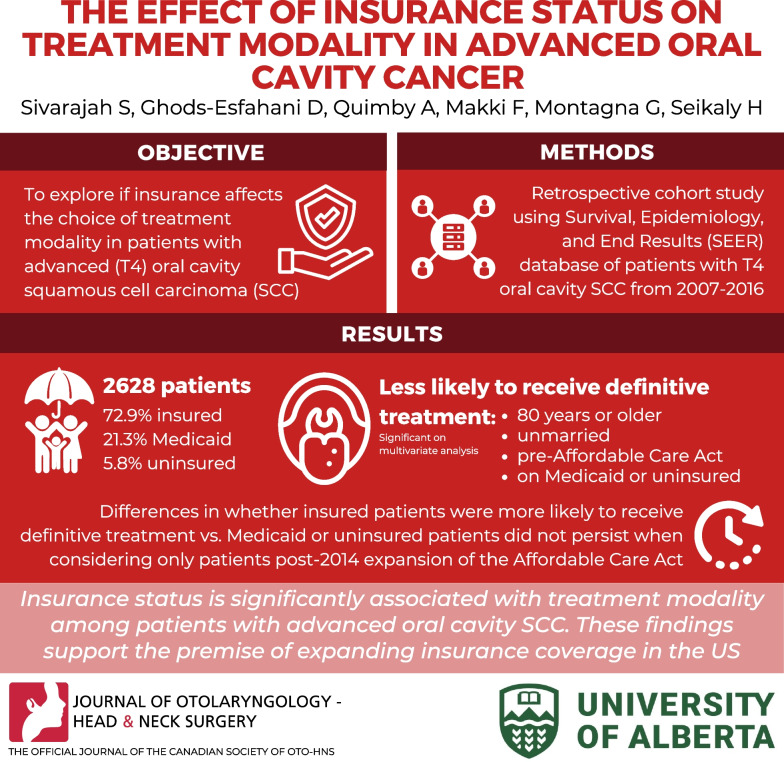

## Background

Head and neck cancers (HNCA) account for 4% of all newly diagnosed cancers each year in the United States [1]. Squamous cell carcinoma of the oral cavity (OCSCC) and oropharynx comprise the majority of HNCA, with a combined incidence of 3% per year [2]. For OCSCC, surgery is generally understood to provide superior oncologic outcomes compared to the primary treatment modalities of radiotherapy and/or chemotherapy. Underscoring this, the National Comprehensive Cancer Network (NCCN) guidelines recommend primary surgery as the first-line treatment modality for OCSCC of all stages (I-IVA), often followed by adjuvant radiation with or without chemotherapy [3]. Non-curative treatment options that do not include surgery are reserved for cases of unresectable disease (stage IVB).

Previous studies have shown that for several different cancer populations in the United States, insurance status impacts cancer stage at initial presentation, with uninsured or publicly insured (Medicare, Medicaid) patients presenting with more advanced cancers than privately insured patients [4–7]. Subsequently, cancer patients who are uninsured have significantly decreased survival outcomes when compared to patients with private insurance [8]. This disparity is most likely multifactorial; however, it is known that insurance type is strongly associated with the odds of receiving definitive treatment with curative intent [9]. This disparity in cancer care and survival is likely even more pronounced for those with advanced stages of disease. To our knowledge, the association between insurance status (including Medicaid coverage), and receipt of definitive treatment has yet to be investigated for patients with OCSCC.

This information is important to help guide public health initiatives that seek to reform access to cancer care, and to assist those with less financial means. The 2010 Patient Protection and Affordable Care Act (ACA) was designed to expand access to healthcare, largely through Medicaid expansion. It has assisted millions of individuals with incomes near the national poverty levels to gain health insurance. However, its impact on extending oncologic care for patients with OCSCC has yet to be rigorously investigated.

Therefore, the aim of this study was to evaluate the association between insurance status and the odds of receiving definitive, guideline-recommended treatment in patients with advanced (T4) OCSCC. We additionally sought to assess whether any potential association between insurance status and treatment type changed subsequent to the introduction of the ACA. We hypothesized that patients with advanced stage disease who were uninsured or were covered by Medicaid would have lower odds of receiving definitive treatment compared to insured patients. We further hypothesized that improvement in such disparities may be appreciable after the 2014 widespread ACA expansion.

## Methods

### Study design

Institutional Review Board exemption was received from the Health Research Ethics Board of Alberta- Cancer Committee. A retrospective, population-based, cohort study was conducted using the National Cancer Institute’s Survival, Epidemiology, and End Results Program (SEER) database. SEER collects population-based data on the incidence, prevalence, and survival of various cancers in the United States. It captures 28% of the US population, and is representative of its geographic, racial and ethnic diversity.

All adult (age ≥ 18) patients with advanced (T4a or T4b) OCSCC diagnosed from 2007 to 2016 were included. 2007 was chosen as a starting point for our population based on the timing of SEER’s collection of information on patient insurance status. Patients were identified based on combinations of variables including tumor histology -squamous cell carcinoma and its variants (basaloid, papillary, spindle cell, verrucous), primary site – mucosal lips (C000–C006, C008–C009); oral tongue (C020–C024, C028–C029); floor of mouth (C040–C049); and gum and other mouth (C030–C039, C050–C059, C060–C069)], and stage- T4, T4a, and T4b. Tumors of the base of tongue and any subsite that included or overlapped with the oropharynx were excluded to prevent confounding or effect modification by human papillomavirus virus (HPV) infection. The following exclusion criteria were used: patients with unknown demographic information including sex, age at diagnosis, race, and marital status; patients with unknown clinical information including histologic grade, AJCC Tumor, Node, Metastasis (TNM) stage; patients with no information on definitive surgery or radiotherapy; and patients with metastatic disease at initial presentation.

### Exposure, outcome, and covariates

SEER variables for insurance and age were combined to create a new 3-level categorical variable of insurance status, defined as: (1) Insured; (2) Medicaid; and (3) Uninsured. The Insured category comprised patients within the following levels of the SEER insurance variables: (1) Private insurance: fee-for-service; (2) Private Insurance: Managed care, HMO or PPO, TRICARE; (3) Medicare- Administered through a Managed Care plan; (4) Medicare with private supplement; (5) Medicare with supplement, NOS and Military; and (6) Insured, No Specifics. The Medicaid category included patients within the following levels of the SEER insurance and age variables: (1) Indian/Public Health Service; (2) Medicaid; (3) Medicaid- Administered through a Managed Care plan; (4) Medicare with Medicaid eligibility; and 5) age ≥ 65 years or greater and are “uninsured” or “unknown”. The Uninsured category was comprised of: (1) Not insured; (2) Not insured, self-pay; and age < 65 years. Insurance status was not available for 35 patients (1.33%). These patients were excluded from our analysis. Our outcome variable, treatment type, was dichotomized as definitive (primary surgery with or without adjuvant treatment) and non-definitive treatment (radiotherapy ± chemotherapy without primary surgery). Data on clinically relevant covariates was also extracted, including age at diagnosis (categorized as 19–29, 30–59, 60–79, 80 + years), sex, year of diagnosis, marital status (married, unmarried, separated, divorced, widowed, unknown), race (defined as White; Black; American Indian, Native American, or Hawaiian; Chinese; Japanese; Filipino; Asian Indian or Pakistani; other; unknown), and oral cavity subsite. Year of diagnosis was categorized as pre- (2007–2013) and post-ACA (2014–2016).

### Statistical analysis

Population demographics were compared across exposure groups using the independent-samples *t*-test and *χ*^2^ test, as appropriate. Univariable logistic regression analysis was conducted to assess the relationship between insurance status and treatment type [10]. A multivariate logistic regression model was also used to determine this association. The model was adjusted for age, sex, year of diagnosis, marital status, race, and primary site. Given a low level of missingness for all covariates, missing data was handled using the complete case method. Sensitivity analyses were performed to assess the effect of cancer stage (T4a or T4b) and oral cavity subsite. Sensitivity analyses were also performed to assess the effect of ACA adoption (year of diagnosis 2007–2013 versus 2014–2016). Outcome measures were reported as odds ratios (ORs) and associated 95% confidence intervals (95% CIs). A *p*-value of < 0.05 was set as the cut-off for statistical significance. All statistical analyses were performed using Stata software, v 15.1 (Stata Corp, College Station, Texas).

## Results

Our study population comprised 2628 patients with OCSCC, of whom 1915 (72.9%) had private insurance (“Insured”), 561 (21.4%) were insured through Medicaid, and 152 (5.8%) were uninsured. Insured patients were more likely to be male (*p* = 0.03), of older age at presentation (*p* < 0.0001), married (*p* < 0.0001), and White (*p* < 0.0001) (Table 1).

**Table 1:** Characteristics of Patients Aged 18 Years and Above with a Diagnosis of Advanced Stage (T4) Oral Cavity Squamous Cell Carcinoma, by Insurance Status, 2007 to 2016

Characteristic	Total	Insured	Medicaid	Uninsured	*P*-value
	*n* = 2,628	*n* = 1,915	*n* = 561	*n* = 152	
Sex, *n* (%)					
Male	1,670 (63.55)	1,196 (62.45)	368 (65.60)	106 (69.74)	0.03
Female	958 (36.45)	719 (37.55)	193 (34.40)	46 (30.26)	
*Age, n* (%)					
18–29	12 (0.47)	8 (0.43)	2 (0.36)	2 (1.32)	< 0.0001
30–59	889 (34.63)	483 (26.00)	294 (52.78)	112 (73.68)	
60–79	1,241 (48.34)	997 (53.66)	206 (36.98)	38 (25.00)	
> 80	425 (16.56)	370 (19.91)	55 (9.87)	–	
Age Mean, (SD)	66 (13.59)	68.39 (13.34)	61.27 (12.54)	53.39 (7.69)	
*Year of diagnosis*					
2007	179 (6.81)	131 (6.84)	35 (6.24)	13 (8.55)	0.06
2008	231 (8.79)	161 (8.41)	57 (10.16)	13 (8.55)	
2009	270 (10.27)	199 (10.39)	54 (9.63)	17 (11.18)	
2010	280 (10.65)	208 (10.86)	49 (8.73)	23 (15.13)	
2011	322 (12.25)	225 (11.75)	76 (13.55)	21 (13.82)	
2012	303 (11.53)	224 (11.70)	55 (9.80)	24 (15.79)	
2013	360 (13.70)	266 (13.89)	77 (13.73)	17 (11.18)	
2014	348 (13.24)	244 (12.74)	92 (16.40)	12 (7.89)	
2015	335 (12.75)	257 (13.42)	66 (11.76)	12 (7.89)	
*Marital status, n* (%)					
Married	1,145 (43.57)	937 (48.93)	162 (28.88)	46 (30.26)	< 0.0001
Unmarried	541 (20.59)	279 (14.57)	198 (35.29)	64 (42.11)	
Separated, Divorced, or Widowed	799 (30.40)	596 (31.12)	166 (29.59)	37 (24.34)	
Unknown	143 (5.44)	103 (5.38)	35 (6.24)	5 (3.29)	
*Race, n* (%)					
White	2,090 (79.53)	1,593 (83.19)	392 (69.88)	105 (69.08)	< 0.0001
Black	317 (12.06)	176 (9.19)	102 (18.18)	39 (25.66)	
American Indian, Native American, or Hawaiian	34 (1.29)	19 (0.99)	15 (2.67)	–	
Chinese	23 (0.88)	14 (0.73)	9 (1.60)	–	
Japanese	27 (1.03)	26 (1.36)	1 (0.18)	–	
Filipino	20 (0.76)	16 (0.84)	3 (0.53)	1 (0.66)	
Asian Indian or Pakistani	64 (2.44)	41 (2.14)	20 (3.57)	3 (1.97)	
Other	51 (1.94)	28 (1.46)	19 (3.39)	4 (2.63)	
Unknown	2 (0.08)	2 (0.10)	–	–	
*Primary site, n* (%)					
Mucosal lips	18 (0.68)	14 (0.73)	4 (0.71)	–	< 0.0001
Oral tongue	763 (29.03)	522 (27.26)	182 (32.44)	59 (38.82)	
Alveolar ridge	634 (24.12)	527 (27.52)	89 (15.86)	18 (11.84)	
Floor of mouth	604 (22.98)	392 (20.47)	166 (29.59)	46 (30.26)	
Palate	195 (7.42)	161 (8.41)	29 (5.17)	5 (3.29)	
Buccal mucosa	226 (8.60)	154 (8.04)	57 (10.16)	15 (9.87)	
Overlapping or NOS	188 (7.15)	145 (7.57)	34 (6.06)	9 (5.92)	
*T Stage*					
T4a	2,365 (88.88)	1,709 (89.24)	496 (88.41)	131 (86.18)	< 0.0001
T4b	227 (8.53)	152 (7.94)	53 (9.45)	19 (12.50)	
T4 NOS	69 (2.59)	54 (2.82)	12 (2.14)	2 (1.32)	
Treatment					
Definitive	1,015 (38.62)	772 (40.31)	191 (34.05)	52 (34.21)	0.014
Non-definitive	1,613 (61.38)	1,143 (59.69)	370 (65.95)	100 (65.79)	

**Fig. 1:**
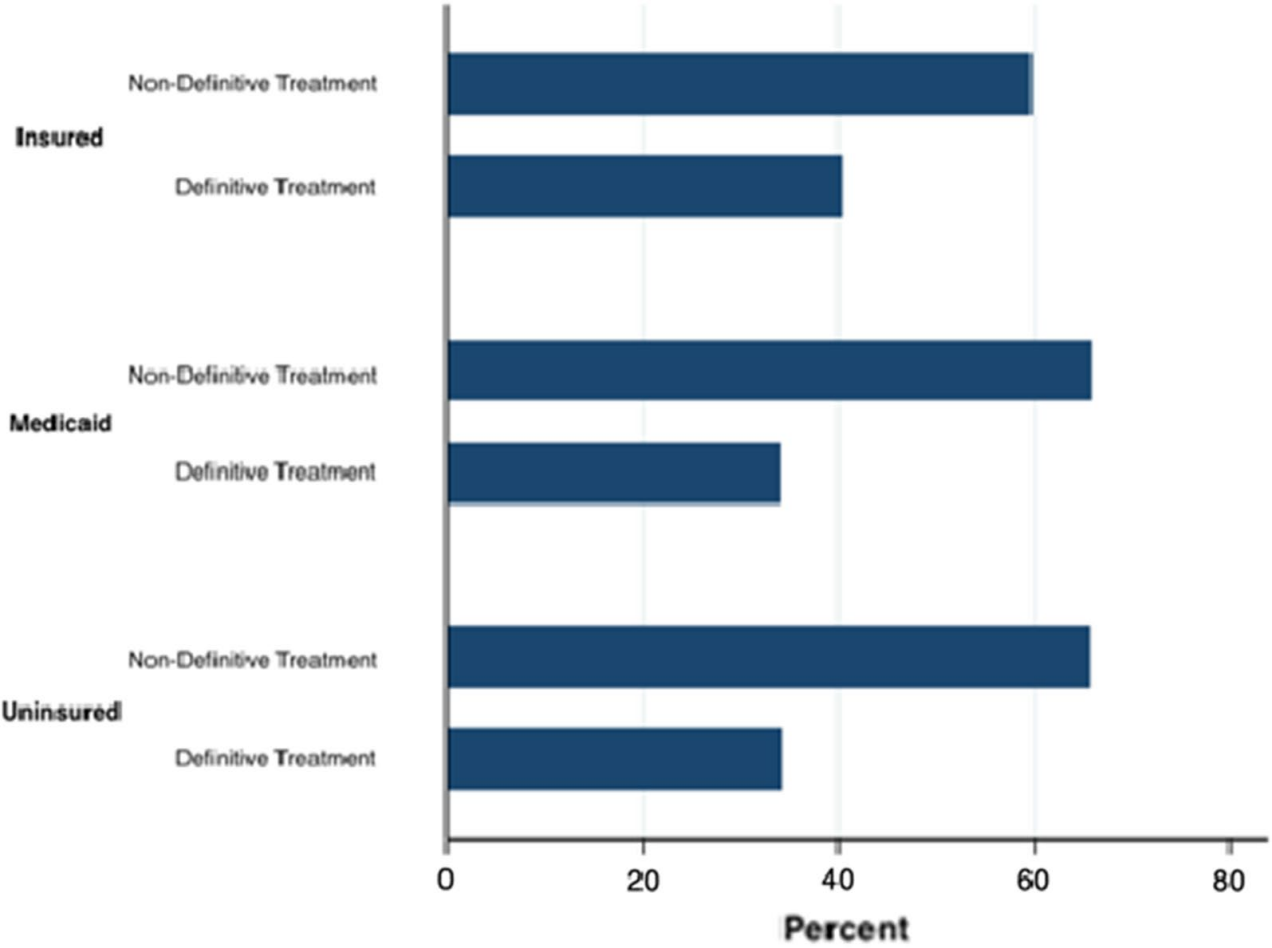
Distribution of definitive and non-definitive treatment types among Insured, Medicaid, and Uninsured insurance categories for patients with T4 oral cavity cancer

**Fig. 2:**
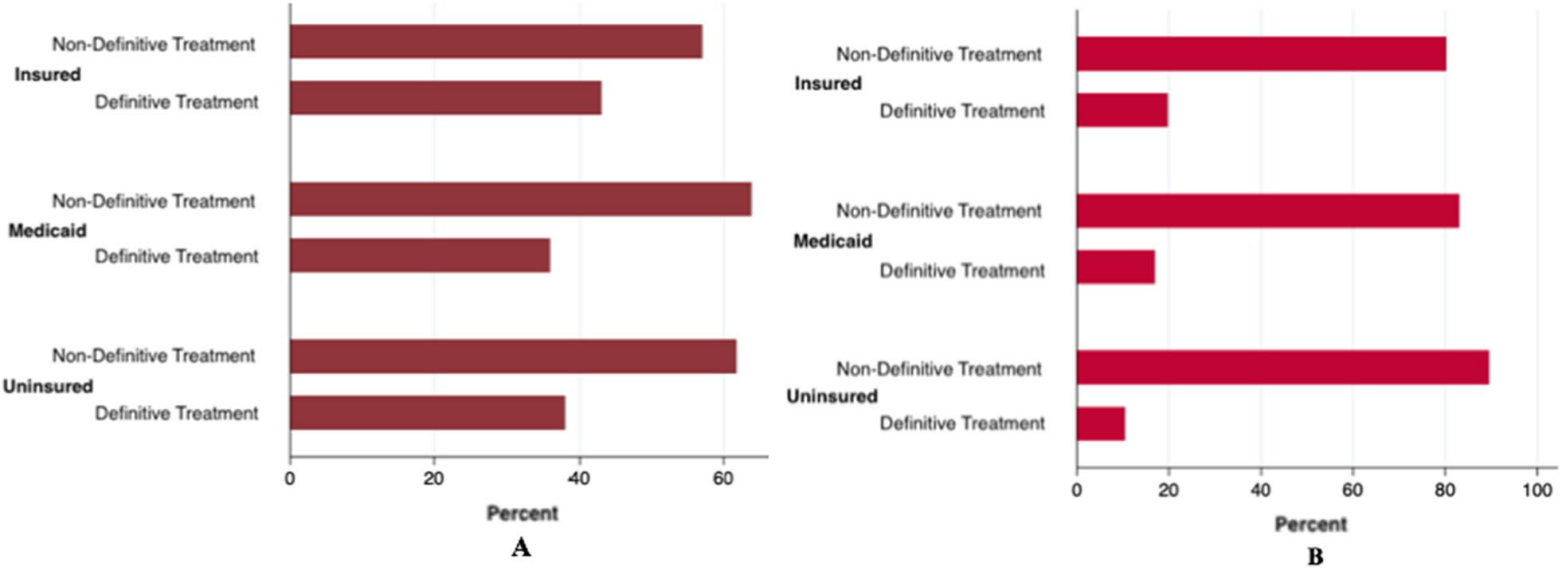
**A** and **B** Distribution of definitive and non-definitive treatment types among Insured, Medicaid, and Uninsured insurance categories for patients with T4a oral cavity cancer (Fig. 2A) and T4b oral cavity cancer (Fig. 2B)

Uni-variable analysis demonstrated that patient characteristics associated with significantly lower odds of receiving definitive treatment included female sex (OR = 0.77; 95% CI 0.66–0.91, *p* = 0.002); unmarried (OR = 0.66; 95% CI 0.53–0.81, *p* < 0.0001); separated, divorced, or widowed (OR = 0.56; 95% CI 0.47–0.69, *p* < 0.0001); T4b disease (OR = 0.31; 95% CI 0.22–0.44, *p* < 0.0001); and Medicaid (OR = 0.76; 95% CI 0.63–0.93, *p* = 0.008) (Table 2). Figure 1 further demonstrates the unadjusted distribution of definitive and non-definitive treatment types among the Insured, Medicaid and Uninsured treatment categories, illustrating that patients who were Uninsured or on Medicaid are less likely to receive definitive surgical treatment (Fig. 2).

**Table 2:** Univariable Analysis with Odds of Receiving Definitive Treatment Among Patients Aged 18 Years and Above with a Diagnosis of Advanced Stage (T4) Oral Cavity Squamous Cell Carcinoma, 2007 to 2016

	OR (95% CI)	*P* value
*Sex, n* (%)\		
Male	1	0.002
Female	0.77 (0.66 – 0.91)	
*Age, n* (%)		
18–29	1	8 (0.43)
30–59	1.78 (0.53 – 5.97)	0.35
60–79	1.35 (0.40 – 4.51)	0.63
> 80	0.44 (0.13 – 1.48)	0.18
*Year of diagnosis*		
2007	1	
2008	1.05 (0.70 – 1.59)	0.81
2009	1.06 (0.71 – 1.58)	0.77
2010	1.35 (0.92 – 2.00)	0.13
2011	1.34 (0.92 – 1.96)	0.13
2012	1.72 (1.17 – 2.52)	0.006
2013	1.18 (0.81 – 1.72)	0.4
2014	1.39 (0.95 – 2.02)	0.09
2015	1.70 (1.17 – 2.48)	0.006
*Year of diagnosis (categorized)*		
< 2014	1	
≥ 2014	1.23 (1.03–1.46)	0.02
*Marital status, n* (%)		
Married	1	
Unmarried	0.66 (0.53 – 0.81)	< 0.0001
Separated, Divorced, or Widowed	0.57 (0.47 – 0.69)	< 0.0001
*T Stage*		
T4a	1	
T4b	0.31 (0.22 – 0.44)	< 0.0001
*Race, n* (%)		
White	1	
Black	0.83 (0.65 – 1.06)	0.13
American Indian, Native American, or Hawaiian	1.07 (0.54 – 2.10)	0.86
Chinese	1.74 (0.77 – 3.97)	0.19
Japanese	1.48 (0.69 – 3.17)	0.31
Filipino	1.31 (0.54 – 3.17)	0.55
Asian Indian or Pakistani	1.80 (1.10- 2.94)	0.02
Other	0.95 (0.53 – 1.68)	0.86
*Primary site, n* (%)		
Mucosal lips	1	
Oral tongue	0.93 (0.34 – 2.50)	0.88
Alveolar ridge	1.87 (0.69 – 5.05)	0.22
Floor of mouth	1.54 (0.57 – 4.17)	0.39
Palate	1.05 (0.38 – 2.91)	0.93
Buccal mucosa	0.95 (0.34 – 2.64)	0.93
Overlapping or NOS	0.87 (0.31 – 2.44)	0.79
*Insurance*		
Insured	1	
Medicaid	0.76 (0.63 – 0.93)	0.008
Uninsured	0.77 (0.54 – 1.09)	0.14

**Fig. 3:**
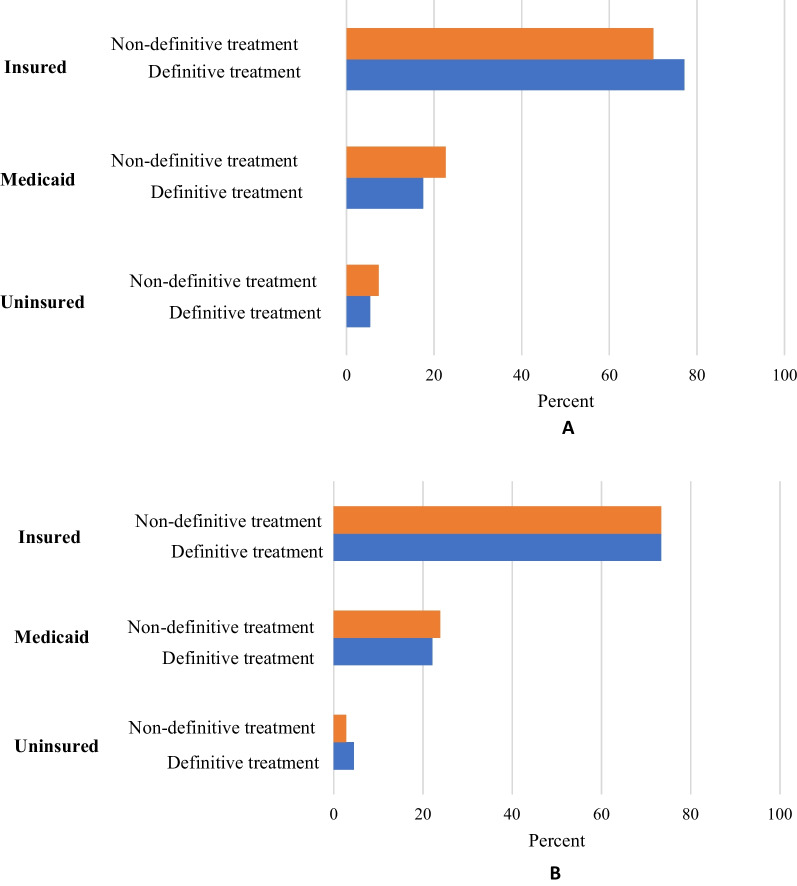
**A** and **B** Distribution of definitive and non-definitive treatment types among Insured, Medicaid, and Uninsured insurance categories for patients before and after the introduction of the Affordable Care Act (ACA)

Patients during the post-ACA period were more likely to receive definitive treatment (OR = 1.22, 95% CI 1.03–1.46, *p* = 0.02) compared to those who received treatment pre-ACA. 37.3% of patients received definitive treatment during the pre-ACA period, which increased to 42.2% of patients in the post-ACA period. For patients insured through Medicaid, while controlling for sex, age, marital status, race, and primary tumor site, those who received treatment pre-ACA were significantly less likely to receive definitive treatment compared to patients with private insurance (OR = 0.59, 95% CI 0.46–0.77, *p* < 0.0001). This disparity was no longer statistically significant in the post-ACA period (OR = 0.81, 95% CI 0.53–1.25, *p* = 0.35). Controlling for the same variables, uninsured patients who received treatment pre-ACA were also significantly less likely to receive definitive treatment compared to patients with private insurance (OR = 0.48, 95% CI 0.31–0.73, *p* = 0.001). Post-ACA, uninsured patients were more likely to receive definitive treatment, although this effect was not statistically significant (OR = 1.36, 95% CI 0.56–3.28, *p* = 0.49). Compared to uninsured patients, patients on Medicaid in the post-ACA period were less likely to receive definitive treatment, although this effect was also not statistically significant (OR = 0.58, 95% CI 0.24–1.45, *p* = 0.32). These differences are illustrated in Fig. 3.

After adjusting for sex, age, year of diagnosis, marital status, race and primary tumor site in the multivariable analysis, patients who were 80 years or older (OR = 0.27; 95% CI 0.07–0.89, *p* = 0.03), with T4b disease (OR = 0.32; 95% CI 0.22–0.47, *p* = 0.04), who were unmarried (OR = 0.67; 95% CI 0.53–0.85, *p* = 0.002), separated, divorced, or widowed (OR = 0.73; 95% CI 0.59–0.90, *p* = 0.004), who received treatment in the pre-ACA period (OR = 0.77; 95% CI 0.64–0.94, *p* = 0.03) and who were on Medicaid (OR = 0.70; 95% CI 0.55–0.88, *p* < 0.0001) or uninsured (OR = 0.63; 95% CI 0.43–0.92, *p* = 0.004) were significantly less likely to receive definitive treatment (Table 3). Insurance status was significantly associated with the odds of receiving definitive treatment only among patients with T4a disease (OR = 0.69, 95% CI 0.54–0.88, *p* = 0.01 [Medicaid vs. Insured]); and OR = 0.65, 95% CI 0.43–0.98, *p* = 0.02 [Uninsured vs Insured]). This disparity was not found for those who presented with T4b disease (OR = 0.36; 95% CI 0.12–1.15, *p* = 0.43 [Medicaid vs. Insured]; and OR = 0.17, 95% CI 0.03–1.04, *p* = 0.82 [Uninsured vs Insured]). Figure 2 shows the crude distribution of treatment types among insurance categories, stratified by T-stage.

**Table 3:** Multivariable Analysis with Odds of Receiving Definitive Treatment Among Patients Aged 18 Years and Above with a Diagnosis of Advanced Stage (T4) Oral Cavity Squamous Cell Carcinoma, 2007 to 2016

	OR (95% CI)	*P* value
*Sex, n* (%)		
Male	1	
Female	0.97 (0.80 – 1.19)	0.82
*Age, n* (%)		
18–29	1	
30–59	1.65 (0.47 – 5.72)	0.43
60–79	1.00 (0.29 – 3.46)	1
> 80	0.27 (0.08 – 0.97)	0.045
*Year of diagnosis*		
2007	1	
2008	1.01 (0.64 – 1.59)	0.97
2009	1.03 (0.66 – 1.59)	0.91
2010	1.15 (0.75 – 1.77)	0.53
2011	1.38 (0.90 – 2.11)	0.1
2012	1.59 (1.04 – 2.44)	0.03
2013	1.03 (0.68 – 1.57)	0.88
2014	1.35 (0.89 – 2.05)	0.16
2015	1.71 (1.13 – 2.61)	0.01
*Year of diagnosis (categorized)*		
< 2014	1	
≥ 2014	1.29 (1.07–1.56)	0.02
Unmarried	0.67 (0.53 – 0.85)	0.001
Separated, Divorced, or Widowed	0.73 (0.59 – 0.90)	0.004
*T Stage*		
T4a	1	
T4b	0.32 (0.22 – 0.47)	< 0.0001

## Discussion

In this nationally representative study of patients diagnosed with advanced but treatable OCSCC (i.e. T4a disease), we found that prior to 2014, patients who were uninsured or with Medicaid insurance were significantly less likely to receive curative-intent surgery than patients with private insurance. The ACA expansion in 2014 seemed to mitigate this disparity for both sets of patients.

Although previous studies have shown disparities in cancer outcomes based on patient insurance status, to the best of our knowledge, this is the first national study to explore the interplay between insurance (including Medicaid coverage) and the receipt of definitive treatment in advanced OCSCC. [8, 9, 11, 12] Expansion of Medicaid coverage, and the provision of subsidies for individuals below the poverty line as legislated by the ACA, is a good first step to addressing the morbidity and mortality associated with OCSCC. These findings underscore the need for ongoing efforts that support equality in the medical care received by different factions of the American population, including those with differing insurance coverage.

It has been previously shown that patients who are uninsured or Medicaid-insured often present with more advanced disease at the time of diagnosis. This increased risk for presenting with late-stage disease has been attributed to a lack of access to screening procedures. For instance, oral cavity cancer is typically detected during routine dental cleanings, and Medicaid covers only limited dental care for patients under the age of 21 [13]. For this reason, we chose to restrict our analysis to patients with advanced stage (T4) OCSCC, to determine whether factors other than advanced stage at presentation contribute to receipt of guideline-recommended treatment. While controlling for stage of disease, our findings suggest that patients with Medicaid insurance in the pre-ACA period were less likely to be offered definitive treatment than those with private insurance.

There are several possible explanations for this observed difference. Firstly, Medicaid patients face barriers to accessing treatment [14]. The relatively lower reimbursement rates of Medicaid insurance are linked to higher rates of physician refusal to provide complex cancer care. The findings of the 2013 National Electronic Health Records Survey is consistent with this notion, and found that the percentage of physicians accepting new patients on Medicaid (68.9%) was much lower than that accepting patients on Medicare (83.7%) and on private insurance (84.7%) [15]. There are several indirect and uncovered costs that can be burdensome for cancer patients. Analysis of commercially-insured individuals revealed that the average medical costs of oral cavity cancers in the first year after diagnosis was $79,151, which is significantly higher than the cost to treat other cancers ($31,559-$65,123) [16–18]. Furthermore, individuals who received multi-modality therapy (surgery, radiation and chemotherapy) averaged $153,892 during the first year after diagnosis [18]. These medical costs are approximately twice any other reported cancer costs. For patients that survived the first year after diagnosis, indirect costs of short-term disability were also high ($7,386 higher) for employees with oral cavity cancer, than for matched employees without cancer [18]. Not all insurance plans are equal; with differences in deductibles, copayments or coinsurance fees, the financial toxicity of cancer can be prohibitive for patients seeking medical care.

The 2014 ACA expansion did seem to reduce the likelihood that uninsured and Medicaid-insured patients would face such prohibitive restrictions to receiving definitive treatment. This is in keeping with recently published reflections on the effects of the ACA on American healthcare, with the country witnessing a substantial decline in the number of uninsured individuals. The ACA expanded eligibility for the Medicaid program to individuals and families with incomes up to 138% of the federal poverty line. The law’s implementation in 2010 has seen the number of uninsured in the country fall by about 20 million [19]. However, it continues to endure numerous legislative challenges following its passage. For instance, the landmark 2012 Supreme Court decision scaled back Medicaid expansion from a nation-wide mandate to a state option.

Other barriers to receiving care include a lack of transportation to medical or dental appointments, the inability to leave work to attend appointments, presence of other comorbidities, treatment at academic versus non-academic hospitals, urban versus rural settings, as well as surgeon case volumes [13, 20]. It is possible that patients who are uninsured or on Medicaid, who are treated at smaller, rural, or non-academic institutions, are less likely to be offered definitive treatment due to a lack of resources or surgeon experience with performing near-total or total glossectomies with advanced reconstruction [21].Further research correlating social determinants of health and individual-level data on socioeconomic factors with cancer care is required for this unique cohort of patients.

Limitations of this study include those inherent to the SEER database. Data on patients’ overall health status and comorbidity burden were unavailable, which may impact surgical candidacy and the decision to pursue definitive treatment. We were not able to control for potentially confounding behavioral risk factors (e.g. smoking and alcohol consumption) and socioeconomic variables (e.g. education level, median household income, and metropolitan status), which may be able to explain at least some of the observed differences amongst treatment type across insurance categories, especially pre-ACA. Finally, this study is also subject to limitations inherent to the use of any large databases, such as the potential for misclassification and coding errors. In most states, individuals who are diagnosed with cancer can qualify for Medicaid, with the eligibility date assigned as the date of diagnosis. Thus, patients can move from the uninsured group to the Medicaid-insured group, confounding the classification of insurance status. In addition, it is also possible that individuals’ insurance coverage may change over the course of their treatment, which may not be captured by SEER.

## Conclusions

Among patients with advanced stage (T4a) OCSCC, insurance status appears to significantly predict the likelihood of receiving definitive cancer treatment. This statistically significant association persists after adjusting for several clinically relevant confounders. Our findings also serve as evidence that healthcare insurance reform- such as through the 2014 ACA- may be an effective means of reducing these inequalities. Further large-scale retrospective or prospective studies should be conducted to confirm the existence of this relationship between insurance status and treatment type, and the effect of Medicaid expansion. Expanding high-quality insurance coverage and understanding the barriers to accessing care is essential for mitigating the morbidity and mortality associated with OCSCC on the US population.

## Data Availability

The datasets used and/or analyzed during the current study are available from the corresponding author on reasonable request.

## Abbreviations

HNCA: Head and neck cancers
OCSCC: Oral cavity squamous cell carcinomas
TNM: Tumor, node, metastasis
OR: Odds ratio
NCCN: National comprehensive cancer network
